# Abnormal brain connectivity in first-episode psychosis: A diffusion MRI tractography study of the corpus callosum

**DOI:** 10.1016/j.neuroimage.2006.12.019

**Published:** 2007-04-01

**Authors:** Gary Price, Mara Cercignani, Geoffrey J.M. Parker, Daniel R. Altmann, Thomas R.E. Barnes, Gareth J. Barker, Eileen M. Joyce, Maria A. Ron

**Affiliations:** a Department of Neuroinflammation, Institute of Neurology, University College London, Queen Square, London, WC1N 3BG, UK; b Imaging Science and Biomedical Engineering, University of Manchester, UK; c Medical Statistics Unit, London School of Hygiene and Tropical Medicine, London, UK; d Imperial College Faculty of Medicine, Charing Cross Campus, London, UK; e King’s College London, Institute of Psychiatry, Department of Clinical Neuroscience, Centre for Neuroimaging Sciences, UK

**Keywords:** DTI, diffusion tensor imaging, FA, fractional anisotropy, MRI, magnetic resonance imaging, MTI, magnetization transfer imaging, PDF, probability density function, PICo, probabilistic index of connectivity, ROI, region of interest, Corpus callosum, Diffusion tensor imaging, First-episode psychosis, Tractography

## Abstract

A model of disconnectivity involving abnormalities in the cortex and connecting white matter pathways may explain the clinical manifestations of schizophrenia. Recently, diffusion imaging tractography has made it possible to study white matter pathways in detail and we present here a study of patients with first-episode psychosis using this technique. We selected the corpus callosum for this study because there is evidence that it is abnormal in schizophrenia. In addition, the topographical organization of its fibers makes it possible to relate focal abnormalities to specific cortical regions. Eighteen patients with first-episode psychosis and 21 healthy subjects took part in the study. A probabilistic tractography algorithm (PICo) was used to study fractional anisotropy (FA). Seed regions were placed in the genu and splenium to track fiber tracts traversing these regions, and a multi-threshold approach to study the probability of connection was used. Multiple linear regressions were used to explore group differences. FA, a measure of tract coherence, was reduced in tracts crossing the genu, and to a lesser degree the splenium, in patients compared with controls. FA was also lower in the genu in females across both groups, but there was no gender-by-group interaction. The FA reduction in patients may be due to aberrant myelination or axonal abnormalities, but the similar tract volumes in the two groups suggest that severe axonal loss is unlikely at this stage of the illness.

## Introduction

Altered connectivity is likely to account for the symptoms and cognitive changes of schizophrenia (Friston and Frith, 1995). A disconnectivity model involving both loss of specialized cortical function and damage to connecting pathways is likely to apply to schizophrenia (Catani and ffytche, 2005). Imaging studies have repeatedly confirmed the presence of cortical abnormalities, especially in prefrontal and temporal heteromodal cortex (Shenton et al., 2001; Bagary et al., 2003; Foong et al., 2001), and synaptic pathology leading to aberrant cortical circuitry has been well documented in neuropathological studies (Harrison and Weinberger, 2005). By comparison, the study of abnormalities in intra- and interhemispheric pathways has received much less attention, although the occurrence of schizophrenia-like symptoms has been documented in diseases involving the white matter (Walterfang et al., 2006).

Among these connecting pathways the corpus callosum is of special interest in schizophrenia. The corpus callosum develops into the third decade of life (Pujol et al., 1993) and the topographical organization of its fibers makes it possible to relate its abnormalities to specific cortical regions. Fibers of small diameter from heteromodal cortex traverse the genu (connecting prefrontal cortex) or the splenium (connecting superior temporal cortex), and larger diameter fibers connecting unimodal motor and sensory cortex traverse the body. A model by Crow (1998), based on the evolutionary aspects of language and the emergence of psychosis in man, postulated that abnormalities in callosal pathways connecting areas of the cortex asymmetrically distributed between the two hemispheres were central to schizophrenia. Since then, conventional MRI studies (Shenton et al., 2001), including those in drug-naive patients with their first episode of schizophrenia (Keshavan et al., 2002), have reported reduction in the size of the corpus callosum, more marked anteriorly, but shape differences in the rostral and mid area of the body containing motor and sensory fibers have also been reported (Goghari et al., 2005). Similar findings have been reported by Bachmann et al. (2003) in a first-episode cohort that included patients with schizophreniform and schizoaffective psychoses. In addition, a longitudinal study of patients with childhood-onset schizophrenia (Keller et al., 2003) has suggested that a failure of normal callosal growth may result in area reductions, particularly in the splenium, by early adulthood. Other imaging studies using voxel-based analysis (Hulshoff Pol et al., 2004) or magnetization transfer imaging (Foong et al., 2001) have also reported abnormalities, predominantly in the genu. In addition Highley et al. (1999a), in a detailed neuropathological study, reported a decrease in axon density in all, but the rostral region of the corpus callosum in female schizophrenics compared to controls. The functional significance of the corpus callosum in schizophrenia has been well documented by studies of interhemispheric transfer of information (Innocenti et al., 2003).

Diffusion tensor imaging (DTI) (Basser et al., 1994) has made it possible to study in vivo the integrity and orientation of neural tissue by measuring water diffusion in the brain. DTI studies have used a region-of-interest (ROI) methodology and have measured the apparent diffusion coefficient (ADC) and/or fractional anisotropy (FA), indices of white matter integrity. An early study from our group (Foong et al., 2000) reported reduced FA in the splenium of the corpus callosum in patients with chronic schizophrenia and similar findings have been reported by others in the splenium (Agartz et al., 2001), or in the structure as a whole (Brambilla et al., 2005; Ardekani et al., 2003; Kubicki et al., 2005). Kumra et al. (2004), on the other hand, failed to find such abnormalities in a small group of patients with early-onset psychosis and the same was the case in the only available study in first-episode patients, also from our group (Price et al., 2005). The clinical correlations of these corpus callosum abnormalities remain uncertain. Brambilla et al. (2005) reported a correlation between DTI changes in the anterior part of the corpus callosum and severity of positive symptoms, but these findings have not been confirmed in other studies.

The information about the direction of diffusion encoded by the eigenvalues and eigenvectors of the diffusion tensor has been used in DTI tractography (Mori et al., 1999) to investigate the continuity of axonal orientation between voxels and thus to infer the paths of fiber tracts in 3 dimensions. DTI tractography has been used in studies of normal subjects (Toosy et al., 2004; Parker and Alexander, 2005; Parker et al., 2005), in patients with multiple sclerosis (Wilson et al., 2003), callosal dysgenesis (Lee et al., 2004) and stroke (Huang et al., 2005). To date only one study (Kanaan et al., 2006) has used tractography to study the corpus callosum in patients with chronic schizophrenia, reporting reduced FA in the genu and it also demonstrated the superiority of this method, over ROI studies, in detecting subtle abnormalities across the whole of a white matter tract.

Here we present, to our knowledge, the first study of the corpus callosum (or indeed any white matter pathway) in patients with first-episode schizophrenia spectrum disorders using diffusion imaging tractography. The main aim of the study was to determine whether subtle abnormalities of interhemispheric connections could be detected using this new technique in a group of patients during the first-episode of psychosis, in whom chronicity-related factors could be excluded. We hypothesized that measures of tract coherence (FA), in white matter traversing the genu and splenium of the corpus callosum, would be significantly reduced in the patient group compared to controls.

## Materials and methods

The patients were recruited as part of a prospective, longitudinal study of first-episode psychosis in West London. Patients were eligible to be recruited into the study if they fulfilled the following criteria; age between 16 and 50 years presenting with a psychotic illness for the first time and less than 12 weeks on antipsychotic medication. Patients eligible for the study were screened using the WHO Psychosis Screen (Jablensky et al., 1992). The diagnosis was established using a structured interview, the diagnostic module of the Diagnostic Interview for Psychosis (DIP, Jablensky et al., 2000), which includes items from the Operational Criteria Checklist for Psychosis (OPCRIT, McGuffin et al., 1991) and the World Health Organization Schedules for Clinical Assessment in Neuropsychiatry (SCAN, Wing et al., 1990). Using these data, a computerized algorithm generates diagnoses under several classification systems including DSM-IIIR and ICD-10. DSM-IIIR diagnoses were subsequently checked against DSM-IV criteria by separately entering OPCRIT items into OPCRIT for Windows (http://sgdp.iop.kcl.ac.uk/opcrit/). Exclusion criteria were the presence of a medical or neurological illness that might impair cognitive function including head injury and alcohol or drug dependency.

Eighteen patients with an initial diagnosis of schizophrenia, schizophreniform or schizoaffective disorder took part in this study. Fifteen of these patients were interviewed again a year later to review the diagnosis, and for the remaining three, who could not be interviewed, a final diagnosis was established also a year later by compiling information from clinicians looking after the patients and by reviewing the clinical notes. Thirteen patients received a final diagnosis of schizophrenia and the remaining five received a diagnosis of schizoaffective disorder (one bipolar, two manic and two depressed subtype). The patients had been ill for a mean of 12.6 months (SD = 19.3 months, range 0–72 months) at entry into the study.

The range and severity of symptoms were assessed at each time point with the Scales for the Assessment of Positive Symptoms (Andreasen, 1983) and Negative Symptoms (Andreasen, 1981), The Young Mania Scale (Young et al., 1978) and the Hamilton Rating Scale for Depression (Hamilton, 1960). The onset of psychosis was established using the Symptom Onset in Schizophrenia inventory (Perkins et al., 2000). Alcohol and drug use was assessed as part of the Diagnostic Interview for Psychosis (see above) and criteria for abuse and dependence were established using the Alcohol Use Scale and the Drug Use Scale (Drake et al., 1990).

The mean age of the patient group was 23.6 years (SD = 6.3; range = 17–38 years), composed of 8 males and 10 females. All patients were receiving antipsychotic medication at the time of scanning. Twenty-one healthy subjects with a mean age of 29.4 years (SD = 7.1; range = 16–42 years) with a gender composition of 6 males and 15 females served as controls. Handedness for all subjects was assessed using the Annett scale (Annett, 1970) as it may be associated with DTI findings in the corpus callosum (Westerhausen et al., 2003). Exclusion criteria were the same as in the patient group as well as history of psychiatric illness in themselves or their first-degree relatives. See Table 1 for a description of study subjects. Permission to conduct the study was obtained from Merton, Sutton and Wandsworth, Riverside and Ealing Research Ethics Committees. All participants gave written informed consent and were paid an honorarium for their time.

### MRI data acquisition

MRI for all subjects was obtained using a GE Signa 1.5 T scanner (General Electric, Milwaukee, WI, USA), which underwent regular quality-control checks, using a standard quadrature head coil. Each subject had a dual-echo fast spin echo scan (TR = 2000 ms, TE_1_/TE_2_ = 19/95 ms, matrix = 256 × 192, field of view [FoV] = 24 × 18 cm^2^), which provides both proton density and T_2_-weighted images. Twenty-eight 5-mm slices were collected, in an oblique-axial plane (parallel to the AC/PC line). An axial plane, IR-SPGR sequence was also performed which obtained a series of 156 contiguous axial slices with the following parameters TE = 5.1 ms, matrix = 256 × 128, field of view = 31 × 16 cm^2^, slice thickness = 1.2 mm, TR = 14.3 ms, flip angle 20°, TI = 450 ms.

Diffusion-weighted single-shot echo planar images (DW-EPI) were acquired in the axial plane (TE = 96 ms, FoV = 22 × 22 cm^2^, matrix = 96 × 96, slice thickness = 2.3 mm, *Δ* = 40 ms, *δ* = 34 ms, resulting in a maximum *b* factor of 1000 s/mm^2^). The acquisition of diffusion-weighted images was gated to the cardiac cycle using a pulse oximeter with a gating scheme optimized for diffusion imaging. Gradients for diffusion sensitization were applied in 54 non-collinear directions. Six images with no diffusion weighting (*b* ≈ 0 s/mm^2^) were also collected for each slice, giving a total of 60 images per slice. Images were interpolated to a 128 × 128 matrix during reconstruction, yielding a final in-plane resolution of 1.72 mm.

### Diffusion processing and analysis

The diffusion tensor was estimated for each voxel according to the method of Basser et al. (1994) and used to compute FA. We also used a model-selection algorithm based on the fit of spherical harmonic series to the diffusion profile (Alexander et al., 2002) to detect the most parsimonious description of diffusion in every voxel. Diffusion within each voxel was classified as isotropic, anisotropic with a single principal direction of diffusion, or anisotropic with more than one direction of diffusion (as in the white matter of the centrum semiovale). The single tensor model was subsequently used for voxels characterized by isotropic diffusion or a single direction of diffusion population. The two-tensor model of diffusion, as described by Parker and Alexander (2003), was used for the remaining voxels.

We used a probabilistic tractography algorithm (PICo or ‘probabilistic index of connectivity’) which considers multiple pathways emanating from a seed point or region (i.e. a group of voxels in a region of interest) (Parker and Alexander, 2003, 2005). Due to the presence of noise in the data, there is some uncertainty associated with the determination of the principal direction of diffusion. The algorithm accounts for this uncertainty by generating a probability density function (PDF) of fiber alignment from the diffusion model of each voxel (which in this case is either the single or the two-tensor model). This provides voxel-wise estimates of confidence in fiber tract alignment, which are then used in the probabilistic tract-tracing procedure. Using the PDFs from a chosen seed point, streamline-based tracking is performed and repeated 10,000 times in a Monte Carlo fashion (sampling each PDF at random on each repeat) to produce tract maps that estimate the probability of connection of every voxel in the brain to a given seed point or region (Parker and Alexander, 2005). Streamlines were propagated using trilinear interpolation of PDFs, as suggested by Behrens et al. (2003), and were terminated if curvature over the scale of a single voxel exceeded 180° or if the path left the brain.

Seed regions were placed in the genu and splenium of the corpus callosum. The genu was defined as the most anterior point of the corpus callosum before it bends downwards and backwards in front of the septum pellucidum, and the splenium as the posterior end of the corpus callosum at its thickest part. Each seed region consisted of a six voxel rectangular shape on a single axial slice. This region size ensured that partial volume effects were completely avoided. Seed regions were placed by displaying the FA maps in three mutually orthogonal orientations using MRIcro (http://www.sph.sc.edu/comd/rorden/) and identifying the sagittal and coronal planes in which the volumes of the genu and splenium were largest. The seed regions were placed on the axial view corresponding to the intersection of these sagittal and coronal planes.

The seed region placement was the only operator-dependent step in the diffusion processing. To assess the inter-rater reliability of the method, a second, independent rater performed the seed region placement in 8 cases, using the same procedure.

The voxel values of the PICo output maps for the corpus callosum range from 0 (no probability of connection) to 1 (certainty of connection) with an intensity resolution step of 0.001 representing the ratio of the number of times that voxel was reached by a streamline originating from the seed point to the total number of iterations in the Monte Carlo process. Each PICo map was thresholded at five probability values ranging from 0.001 (the lowest recorded value of probability of connection) to 0.03, at logarithmically spaced intervals, to generate five objective binary masks (thresholded at 0.001, 0.002, 0.006, 0.013 and 0.03). This multi-threshold approach, previously used in the study of visual pathways (Toosy et al., 2004), allows with increasing degrees of certainty the reconstruction of the core of the tract, where fiber alignment should be greatest, and the exploration of the probability of connection of distant voxels to the seed point. For each threshold the mean tract volume and the mean FA were calculated for each subject.

The spatial variability of the tract across each subject group was characterized as previously described (Toosy et al., 2004; Parker et al., 2005). Firstly the non-diffusion-weighted (*b* = 0) images (inherently co-registered with PICo output images) were normalized to a stereotactic space (Montréal Neurological Institute, MNI) using the standard echo planar image template in SPM2 (Wellcome Department of Cognitive Neurology, Institute of Neurology, London, UK) running in MATLAB (MathWorks, MA). The normalization parameters thus obtained were then applied to the binary images of the tract obtained by thresholding the probability images at the level that maximized the difference in FA between patients and controls (see below). After normalization, these images were averaged on a voxel-by-voxel basis, producing a map used to display the degree of tract overlap between subjects within each group.

In order to detect the presence of ventricular enlargement in the patient group, which might affect tractography, we performed a voxel-based comparison of CSF maps between the two groups using SPM2 (Wellcome Department of Cognitive Neurology, Institute of Neurology, London, UK). The IR-SPGR images were segmented and normalized using the iterative procedure described in Good et al. (2001), thus performing first a segmentation in native space, next normalizing gray matter images using the standard a priori gray matter template available in SPM2 and applying the same normalization parameters to the whole volume. The normalized volume was then segmented again yielding white matter, gray matter and CSF probability images. Voxel values in segmented images were multiplied by the Jacobian determinants derived from spatial normalization (modulation) to provide intensity correction for induced regional volumetric changes, thus preserving within-voxel volumes that may have been altered during non-linear normalization (Ashburner and Friston, 2000). CSF images were also smoothed to 6 mm (Full-Width-Half-Maximum) Gaussian filter. Smoothing is required to accommodate anatomical variation between subjects and therefore results in more normally distributed data. Global CSF volumes in cm^3^ were also obtained by integrating the CSF image signal intensity over the whole volume for each subject.

### Statistical analysis

Age, gender and handedness distributions in the two groups were compared using *t*-tests and *χ*^2^ tests.

The inter-rater reproducibility of the method was tested by comparing the tract volumes obtained by the two raters. Inter-rater reliability was assessed using the coefficient of variation (CoV), CoV = (*σ* / *μ*) × 100%, where *σ* is the standard deviation and *μ* is the mean, with lower values indicating better reproducibility.

Multiple linear regressions of mean FA were carried out to compare tract coherence in patients and controls, with gender, age and tract volume as covariates, to control for between-group differences in these covariates. To avoid spurious positive results due to multiple comparisons, the multiple regressions were carried out simultaneously for each of the five thresholds in the genu and the splenium (ten regressions) using Zellner’s seemingly unrelated regression method (Zellner, 1962), which yields a single significance value (from the *F*-statistic) of the difference between patients and controls across all ten regressions, in addition to individual values for each regression. The *F*-statistic is obtained by comparing the ten regressions obtained including or excluding group membership and taking into account the correlations between the residuals from all ten equations. This method also allows a test of whether region (genu or splenium) or probability thresholds modify the differences in FA between patients and controls. Similar multiple linear regressions were performed to explore gender differences in FA (when adjusting for group membership, age and tract volume) as well as age differences in FA (adjusting for group membership, gender and tract volume).

A voxel-based statistical comparison between CSF maps from patients and controls was performed in SPM2 based on the General Linear Model and Gaussian random field theory incorporating subject age and gender into this model.

## Results

Patients were significantly younger than controls. The mean age for patients was 23.6 years and 29.4 years for controls (*t* = − 2.7, *df* = 37, *P* = 0.011). However, there was an age overlap between the two groups, allowing the results to be adjusted for age. There were no significant differences in gender distribution (*χ*^2^ = 0.62, *df* = 1, *P* = 0.43) or handedness (*χ*^2^ = 1.81, *df* = 1, *P* = 0.18) between the groups. All patients were receiving antipsychotic medication and two were on mood stabilizers at the time of the study (see Table 1). The average duration of treatment prior to scanning was 61 days (range 8–153 days).

The coefficient of variation for the tract volume for the 8 repeated measurements performed by two raters was 1.36%, indicating a very high inter-rater reproducibility.

**Fig. 1:**
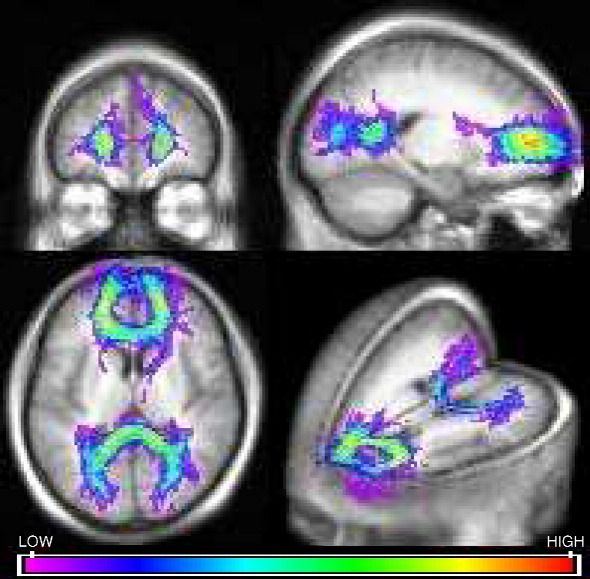
Tract overlap maps of the genu and splenium for the whole patient group displayed in MRIcro. Overlap is greatest in the core of the tract.

**Fig. 2:**
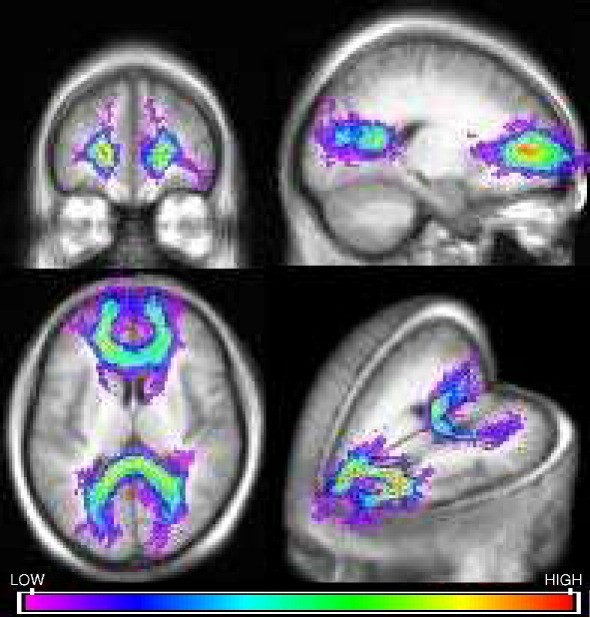
Tract overlap maps of the genu and splenium for the whole control group displayed in MRIcro. Overlap is greatest in the core of the tract.

The tract overlap maps for the genu and splenium in patients and controls (Figs. 1 and 2) were compared with reference maps of the corpus callosum to ascertain their anatomical validity.

**Fig. 3:**
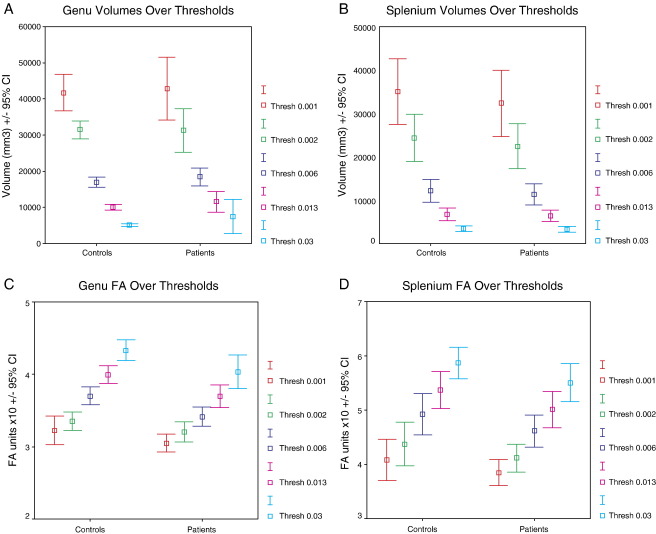
(A) The estimated genu tract volumes compared in the patient and control population at each of the probabilistic thresholds (0.001, 0.002, 0.006, 0.013, 0.03). (B) The estimated splenium tract volumes compared in the patient and control population at each of the probabilistic thresholds (0.001, 0.002, 0.006, 0.013, 0.03). (C) Comparisons of the estimated genu tract FA between patients and controls at each of the probabilistic thresholds (0.001, 0.002, 0.006, 0.013, 0.03). (D) Comparisons of the estimated splenium tract FA between patients and controls at each of the probabilistic thresholds (0.001, 0.002, 0.006, 0.013, 0.03).

The estimated tract volumes in patients and controls decreased at higher connection probability thresholds as the core of the tract was progressively isolated. Conversely, FA increased with higher thresholds (Figs. 3A–D).

The results of the multiple regression analysis comparing FA in patients and controls are shown in Table 2.

The single test of significance considering both regions of the corpus callosum at all thresholds was *P* ≤ 0.02, indicating a significant difference in FA between the two groups in either region, over all thresholds. Inspection of the differences in FA at the individual thresholds indicated that the differences in FA between the groups are due to lower FA in the patient group in the genu at probability thresholds 0.006 and 0.013, and to a lesser extent in the splenium at thresholds 0.013 and 0.03 (see Table 2).

To examine subgroup differences in FA, we performed a regression analysis to compare the 13 schizophrenic and 5 schizoaffective patients. Both subgroups had a lower FA than controls. Results from the regression analysis revealed that in the genu, the schizoaffective group had a lower FA than the schizophrenia group (*P* = 0.0806), whereas in the splenium, the schizoaffective group had higher FA values than the schizophrenia group (*P* = 0.0295). This suggests that, although there may be subtle differences between subgroups, they differed from controls in the same direction and amalgamating the subgroups does not obscure the differences between patients and controls.

We performed a separate analysis looking for gender and age differences in FA by considering patients and controls together. In the analysis of gender, a single test of significance considering all thresholds and both regions of the corpus callosum gave a *P* = 0.0001. This result indicates that there are gender differences in FA irrespective of subject status. Thus FA, adjusted for group membership, was lower in females than males in the genu at thresholds 0.001 and 0.002, but not in the splenium. There was no significant group membership-by-gender interaction (*P* = 0.2).

With respect to the effects of age, there was no evidence that the difference between patients and controls in FA varied with age (*P* = 0.791) for the global test over both regions (i.e. the ten equations), after adjusting for gender and tract volume.

Finally, the SPM analysis using a voxel-based comparison of CSF maps between the two groups revealed no differences between patients and controls using a family wise error correction for multiple comparisons, with *f* = 34.42 and no voxels as an extent threshold. This indicates that no ventricular enlargement was present in the patient group relative to controls. There was no significant difference between the global CSF volumes of patients (287 cm^3^, SD = 33.6) and controls (283 cm^3^, SD = 51.6) (*t* = − 0.85, *P* = 0.40, CI = [− 41.7–17.2]).

## Discussion

Our findings provide evidence of altered interhemispheric connectivity in patients with first-episode schizophrenia spectrum disorders. In the patient group, abnormal tract coherence, as measured by reduction in FA, was present in tracts traversing the genu and, to a lesser extent, in those traversing the splenium. Tract coherence, as measured by FA, was lower in females, both in patients and controls, but we failed to find a gender-by-group interaction.

The genu and the anterior part of the body of the corpus callosum contain mainly connections from prefrontal cortex, cingulate and insula, and pathology in these cortical regions could result in the abnormalities of the corpus callosum described here. Similarly, a decreased thalamic input to the frontal and temporal cortex, perhaps due to excessive synaptic pruning, could also result in decreased cortical connections and hence in abnormalities in the corpus callosum (Innocenti et al., 2003), but it is also possible that primary white matter abnormalities could contribute to our findings (Walterfang et al., 2006). The corpus callosum develops alongside other midline structures, namely the fornix, hippocampus, septum and cingulate cortex, and developmental or maturational abnormalities involving the corpus callosum are also likely to affect these structures known to play a role in schizophrenia.

Our diffusion-MRI-based tractography (PICo) used a probabilistic approach that takes into account branching, crossing and merging of tract fibers and this represents an advantage over previous deterministic approaches that do not take these anatomical features into consideration (Kanaan et al., 2006). In addition the use of different probability thresholds and simultaneous multiple regression analysis (Zellner, 1962) has allowed us to demonstrate evidence, from a single statistical test, that there is some difference in tract coherence between patients and controls and to explore the characteristics of the core of the tract in the genu and the splenium. The group differences in FA become more apparent at intermediate probability thresholds in the genu when fiber alignment in the core of the corpus callosum is greatest and aberrant branching that could have artificially reduced FA (Jones et al., 2005) less likely. The high inter-rater reliability in the placement of the seed points also suggests that the differences in FA between patients and controls are likely to be illness-related rather than artifactual. The same applies to the effects of gender and age, known to affect FA (Price et al., 2005; Jones et al., 2005), that were statistically controlled for in our study as our groups were not closely age matched. Antipsychotic medication may have played a role in reducing FA, although it seems unlikely that medication effects could fully account for the differences between the groups in first-episode patients who had been exposed to medication for short periods. Moreover, others (Flynn et al., 2003) have found no correlation between the presence of white matter abnormalities in first-episode psychosis and exposure to antipsychotic medication. The possible effect of mood stabilizers in explaining our results is likely to be negligible as they were used in only 2 patients.

The gender difference in tract coherence that we found in our subjects, particularly in the genu, supports our previous results in a different sample of first-episode patients and controls using a region-of-interest methodology (Price et al., 2005) and is in keeping with the lower anisotropy in normal females than males reported by Westerhausen et al. (2003). Gender dimorphism and maturational differences are likely to be relevant in explaining this finding. Dubb et al. (2003), in an MRI study of normal subjects, found a larger genu and smaller splenium in men compared with women and attributed the differences to different hormonal influences, to enhanced motor coordination in men (interhemispheric connections crossing the genu) and to greater bihemispheric representation of language in women (tracts crossing the splenium). Adult maturational changes in the corpus callosum, with a peak in the third decade of life, would further accentuate these differences. The differences in age between the two groups are a limitation of the study, although we tried to circumvent the problem using age as a covariate.

The precise neuropathological correlates of FA abnormalities remain to be fully elucidated. Axonal membranes are considered to be the main determinant of anisotropy in neural tissue, and pathological or experimental models of axonal degeneration have lead to reductions in FA. Myelin abnormalities are also responsible for changes in FA, although their contribution may be less important than those of axons or axonal membranes (Beaulieu, 2002). In our study the lower FA in the white matter tracts traversing the corpus callosum may be due, in addition to myelin abnormalities, to differences in axonal membranes, alterations in axonal packing density, mean axonal diameter (for example due to a bias towards axons of greater or lesser diameter in one or other group) or a less coherent fiber alignment in the patient group. The similar tract volumes we found in patients and controls may suggest that severe axonal loss is unlikely at this early stage of the illness. On the other hand, the greater variance in genu tract volumes in the patient group suggests that there may be more branching (i.e. less coherence or alignment) in the core of the tract. We have also excluded the possibility that our findings could be related to ventricular enlargement in the patient group.

The study of patients with first-episode psychosis has the advantage of minimizing the effects of chronicity and lengthy exposure to medication, on the other hand, such patients are diagnostically heterogeneous and this is one of the shortcomings of our study and it remains uncertain whether the changes in corpus callosum described here are a core neuropathological abnormality common to all psychosis or are only present in a subgroup of patients. Support for the former accrues from the study of Bachmann et al. (2003) who reported similar findings in a heterogeneous group of patients with first-episode psychosis. In addition, there is evidence to suggest that corpus callosum abnormalities are also present in patients with bipolar disorder (Brambilla et al., 2003, 2004) and other DTI studies looking at ROIs in frontal white matter, although not specifically at the corpus callosum, have reported low FA in a mixed group of patients with first-episode psychosis (Szeszko et al., 2005) and in those with first-episode mania (Adler et al., 2006). There is also evidence that oligodendrocyte and myelination genes may be downregulated both in schizophrenic and affective psychosis (Tkachev et al., 2003; Iwamoto et al., 2005). It is also likely that the abnormalities described here may also be present in other connecting tracts, as suggested by the neuropathological findings of Highley et al. (1999b) in the anterior commissure, and the results of DTI imaging studies (Kubicki et al., 2005). Our study suggests that the schizophrenia and schizoaffective subgroups may be heterogeneous based on FA, but both these groups differ from controls in the same direction of FA change. An intriguing possibility, in need of further study, is that genetic variability within schizophrenia may be associated with specific patterns of brain morphology including variations in the size and structure of the corpus callosum (Agartz et al., 2006).

Our findings also demonstrate that tractography is capable of detecting subtle pathological abnormalities in patients with psychosis early in the disease that may go undetected using other DTI methods (Price et al., 2005; Kanaan et al., 2006). Tractography is likely to have a role in future psychosis research, in elucidating connections between relevant cortical regions and in giving specific information about white matter abnormalities and their evolution over time.

## Figures and Tables

**Table 1:** Summary of patient and control characteristics

	FE psychosis patients *n* = 18	Controls *n* = 21
Age	23.6 years	29.4 years
Age range	17–38 years	16–42 years
Gender	8 male	6 male
10 female	15 female
Handedness	16 Right	15 right
2 Left	6 left
Diagnosis	13 schizophrenia	–
5 schizoaffective
Antipsychotic medication (18 FE patients)	2 amisulpride	None
1 flupentixol
12 olanzapine
3 risperidone
Antipsychotic medication		–
Duration mean	61 days
Duration range	8–153 days
Duration up to 1 month	7 patients
Duration 1–2 months	6 patients
Duration 2–3 months	1 patient
Duration 3–4 months	3 patients
Duration 4–5 months	0 patient
Duration over 5 months	1 patient
Other medications (2 FE patients)	1 lithium	None
1 lithium and sodium valproate

FE = first-episode.

**Table 2:** Multiple linear regression analysis, exploring the differences in FA between patients and controls, in the genu and splenium at different probability thresholds

FA differences	Group coefficient^a^	Significance (*P*)	95% confidence interval
*Genu*			
Threshold 0.001	− 0.013	0.163	− 0.031 to 0.005
Threshold 0.002	− 0.013	0.144	− 0.031 to 0.005
Threshold 0.006	− 0.021	0.021*	− 0.039 to − 0.003
Threshold 0.013	− 0.022	0.022*	− 0.041 to − 0.003
Threshold 0.03	− 0.016	0.168	− 0.038 to 0.007

*Splenium*			
Threshold 0.001	− 0.026	0.246	− 0.069 to 0.018
Threshold 0.002	− 0.030	0.192	− 0.075 to 0.015
Threshold 0.006	− 0.036	0.122	− 0.081 to 0.010
Threshold 0.013	− 0.040	0.078	− 0.085 to 0.005
Threshold 0.03	− 0.039	0.062	− 0.080 to 0.002

Age, gender and tract volume entered as covariates.

a Group coefficient = mean FA in patients − mean FA in controls, after adjusting for age, gender and tract volume. *P < 0.05.
