# Corrigendum to “Abnormal brain connectivity in first-episode psychosis: A diffusion MRI tractography study of the corpus callosum” [NeuroImage 35 (2007) 458–466]

**DOI:** 10.1016/j.neuroimage.2007.05.064

**Published:** 2007-12

**Authors:** Gary Price, Mara Cercignani, Geoffrey J.M. Parker, Daniel R. Altmann, Thomas R.E. Barnes, Gareth J. Barker, Eileen M. Joyce, Maria A. Ron

**Affiliations:** a Department of Neuroinflammation, Institute of Neurology, University College London, Queen Square, London WC1N 3BG, UK; b Imaging Science and Biomedical Engineering, University of Manchester, UK; c Medical Statistics Unit, London School of Hygiene and Tropical Medicine, London, UK; d Imperial College Faculty of Medicine, Charing Cross Campus, London, UK; e King’s College London, Institute of Psychiatry, Department of Clinical Neuroscience, Centre for Neuroimaging Sciences, UK

We have noticed an error in the “Methods” section. The error, which has *no* impact on the results or conclusions reached in the article, is detailed as follows:

The reported gender composition in the article was 8 males and 10 females for the patient group and 6 males and 15 females for the control group. It should have been reported as 10 males and 8 females for the patient group and 9 males and 12 females for the control group. The reported results for the gender comparison in the “Results” section on page 461, first paragraph, line 4 [“There were no significant differences in gender distribution (χ2 = 0.62, *df* = 1, *P* = 0.43) ”] still remains true, as this analysis was based on the true gender composition of 10 males and 8 females for the patient group and 9 males and 12 females for the control group. All other analyses incorporating gender also incorporate the true gender values.

Finally, in the section “Diffusing processing and analysis” on page 460, paragraph 2, line 20, “180°” should read “80°”.

We apologize for any inconvenience these errors may have caused.

